# Individual and facility level factors influencing timely antenatal care initiation in Ethiopia: A multilevel analysis of the 2021/2022 ESPA data

**DOI:** 10.1371/journal.pone.0334320

**Published:** 2025-10-10

**Authors:** Addisu Alehegn Alemu, Alec Welsh, Theodros Getachew, Marjan Khajehei

**Affiliations:** 1 College of Health Sciences, Debre Markos University, Debre Markos, Ethiopia; 2 School of Women’s and Children’s Health, University of New South Wales, Sydney, New South Wales, Australia; 3 Discipline of Women’s Health, School of Clinical Medicine, University of New South Wales, Sydney, New South Wales, Australia; 4 Department of Maternal-Fetal Medicine, Royal Hospital for Women, Randwick, New South Wales, Australia; 5 Health System Research Directorate, Ethiopian Public Health Institute, Addis Ababa, Ethiopia; 6 Department of Global Health and Population, Harvard T.H. Chan School of Public Health, Takemi Program in International Health, Boston, Massachusetts, United States of America; 7 Women’s and Newborn Health, Westmead Hospital, Westmead, New South Wales, Australia; 8 The University of Sydney, Sydney, New South Wales, Australia; 9 Western Sydney University, Sydney, New South Wales, Australia; Wolaita Sodo University College: Wolaita Sodo University, ETHIOPIA

## Abstract

**Background:**

Antenatal care (ANC) is a package of healthcare services for pregnant women that improves the health of both the women and their unborn babies, with a minimum of eight times and most effective when initiated within the first 12 weeks. This study aimed to assess the magnitude and factors associated with timely ANC initiation among pregnant women attending their first ANC visit during their most recent pregnancy in Ethiopia.

**Methods:**

We analysed weighted data from 2,037 pregnant women who attended their first ANC visit during their most recent pregnancy, extracted from the 2021/2022 Ethiopian Service Provision Assessment. We fitted a multilevel mixed-effects logistic regression model to examine individual and facility level factors influencing timely ANC initiation, which was calculated in weeks. We reported descriptive statistics using frequencies and percentages, and regression results using adjusted odds ratios (AORs) with 95% confidence intervals (CIs). We conducted all analyses using STATA version 16 software.

**Results:**

The magnitude of timely ANC initiation in Ethiopia was 14.9%, with a mean gestational age at initiation of 22 weeks. Primary education (AOR = 1.74, 95% CI: 1.08, 2.82), partner involvement (AOR = 1.42, 95% CI: 1.03, 1.96), and attending non-public facilities (AOR = 2.49, 95% CI: 1.45, 4.28) were associated with higher odds of timely ANC initiation. Conversely, bypassing nearby facilities (AOR = 0.44, 95% CI: 0.30, 0.64) and attending facilities in large central regions (AOR = 0.24, 95% CI: 0.15, 0.40) or small peripheral regions (AOR = 0.27, 95% CI: 0.13, 0.52) were associated with lower odds of timely ANC initiation.

**Conclusion:**

One in seven pregnant women in Ethiopia initiated ANC within the first 12 weeks of pregnancy, far below the World Health Organization recommendation that all pregnant women should begin ANC within this period. Timely ANC initiation could be explained by factors related to women, their partners, and healthcare facilities. Policy interventions should prioritise women’s education, partner involvement, utilisation of nearby available healthcare facilities, community awareness to improve timely ANC initiation in Ethiopia.

## Background

Maternal and neonatal mortality has long been a global public health issue and remains agenda to the Sustainable Development Goals (SDGs) (2015–2030) [1–3]. SDG targets 3.1 and 3.2 aim to reduce global maternal mortality to below 70 deaths per 100,000 live births and neonatal mortality to below 12 deaths per 1,000 live births by 2030 [2]. Yet, projections showed that global maternal mortality will remain at 167 per 100,000 live births, and 42 of 48 countries in Sub-Saharan Africa (SSA) are unlikely to achieve the neonatal mortality reduction target [4,5]. SSA continues to have the highest maternal and neonatal mortality worldwide, with 454 maternal deaths per 100,000 live births and 27 neonatal deaths per 1,000 live births [6,7]. Within the region, Ethiopia is among the countries with high maternal and neonatal mortality, with 267 maternal deaths per 100,000 live births and 33 neonatal deaths per 1,000 live births [8–10]. The leading causes of maternal death in Ethiopia are haemorrhage, uterine rupture, and pregnancy-induced hypertension, accounting for 29.9%, 22.3%, and 16.9% of maternal deaths, respectively [11] while nearly 90% of neonatal mortality is due to prematurity, sepsis, and birth-related complications [12]. The World Health Organization (WHO) emphasises that most maternal and neonatal deaths are preventable through timely and appropriate healthcare services, with antenatal care serving as a cornerstone of these efforts [7,13].

Antenatal care (ANC) refers to a mosaic of healthcare services delivered by skilled professionals to pregnant women from conception to childbirth, aimed at ensuring optimal health for both mothers and their unborn babies [14]. ANC includes the assessment of health risks, the provision of health education and promotion, the prevention and management of potential complications during pregnancy, and the identification of pregnancies at risk of complications during childbirth [14,15]. These create opportunities for pregnant women to receive immunisations such as tetanus, micronutrients including iron, screening and treatment for illnesses such as anaemia, and for infectious diseases such as HIV, including testing and prevention of mother to child transmission, all are essential for maternal and neonatal health [16]. Consequently, ANC is recognised as one of the most effective interventions worldwide to end preventable maternal and neonatal deaths and improve pregnancy outcomes [17,18].

ANC used to be difficult to utilise, particularly in developing countries, until the WHO introduced a universal guideline in 2002 that specified its initiation time and the interventions with their respective frequencies, based on those proven to improve maternal and neonatal health [19]. The guideline advised that all pregnant women attend a minimum of four ANC visits, with the first occurring within the first 12 weeks of pregnancy, which increased ANC utilisation, especially in developing countries [14]. In 2016, the WHO revised the guideline, increasing the recommended number of ANC visits from four to eight, following evidence demonstrating further reductions in maternal and neonatal mortality [14]. The revision emphasises the pressing need to prioritise women who delay ANC initiation, as they are less likely to follow the guideline for achieving optimal pregnancy outcomes [20]. Initiating ANC within the first 12 weeks of pregnancy, referred to as timely ANC initiation, is crucial for ensuring women adhere to the guideline and allows providers to prevent, diagnose, and manage potential complications associated with pregnancy [20–22].

However, timely ANC initiation remains low, especially in low-income countries (LMICs), despite 88% of women worldwide could utilise at least one ANC in 2023 [16]. For instance, a study conducted in LMICs in 2020 showed that among the 49.9% of women who utilised at least one ANC, 44.3% initiated ANC timely [20]. Similarly, a recent study in SSA reported that 38% of women-initiated ANC timely, compared with 32.7% of women in Ethiopia [21]. This highlights that the disparity between high-income countries and LMICs exists not only in the timing of ANC initiation but also in the overall utilisation of ANC service. Timely ANC initiation also varies across Ethiopia, ranging from 29% in the Ilu Ababor Zone [23] to 42% in Southwest Ethiopia [24].

Nationwide studies in Ethiopia reported that 32.7% to 37.7% of women initiated ANC timely, with several factors positively associated with timely initiation, including higher maternal and partner education, greater economic status, urban residence, intended pregnancy, younger maternal age, maternal employment, nulliparity, maternal knowledge of ANC, pregnancy complications, previous facility birth, being married, and a family size of fewer than five [25–30]. However, these studies were community-based and relied on women’s recall in months to estimate the timing of ANC initiation, which could introduce recall bias and limit the accuracy of the estimates [20]. In addition, the studies did not assess healthcare facility related factors that could influence women’s timely ANC initiation.

In this study, we assessed the magnitude and individual- and facility-level factors influencing timely ANC initiation (in weeks) among pregnant women attending healthcare facilities for ANC for the first time during their most recent pregnancy, extracting data from the most recent nationwide Ethiopian Service Provision Assessment (ESPA) survey. The findings are expected to inform strategies to improve timely ANC initiation and provide a foundation for monitoring progress toward achieving WHO recommendations and maternal and neonatal mortality related SDGs.

## Methods

### Data source

We used the most recent ESPA survey in Ethiopia, conducted from August 11, 2021, to February 4, 2022. The survey was a cross-sectional, facility-based study and was the second nationwide assessment of healthcare facilities in the country. The ESPA was carried out by the Ethiopian Public Health Institute in collaboration with the Ethiopian Ministry of Health, with support from the United States Agency for International Development (USAID). This survey utilised standardized tools for cross-country comparisons, as well as sufficient sample size for sub-national and facility-specific estimates [31,32].

The tools included three questionnaires and an observation checklist: The facility inventory questionnaire assessed facility readiness for priority services including ANC [33]; the healthcare provider questionnaire gathered data such as providers’ qualifications, supervision, and perceptions of the service environment [34]; the observation checklist documented consultations, including information exchange during procedures [35]; and the client exit interview questionnaire evaluated client understanding and attitudes toward the service they received [36].

Furthermore, the ESPA data were collected by experienced data collectors who underwent four weeks of intensive training, including pretesting of the tools [37]. We obtained publicly available, de-identified data from the Demographic and Health Survey (DHS) website (www.dhsprogram.com) following a formal request and after receiving ethical approval. All methods adhered to the designated protocols outlined in the ESPA survey. The study followed the Strengthening the Reporting of Observational Studies in Epidemiology (STROBE) guidelines for cross-sectional studies.

### Sample size and sampling procedure

**Fig 1:**
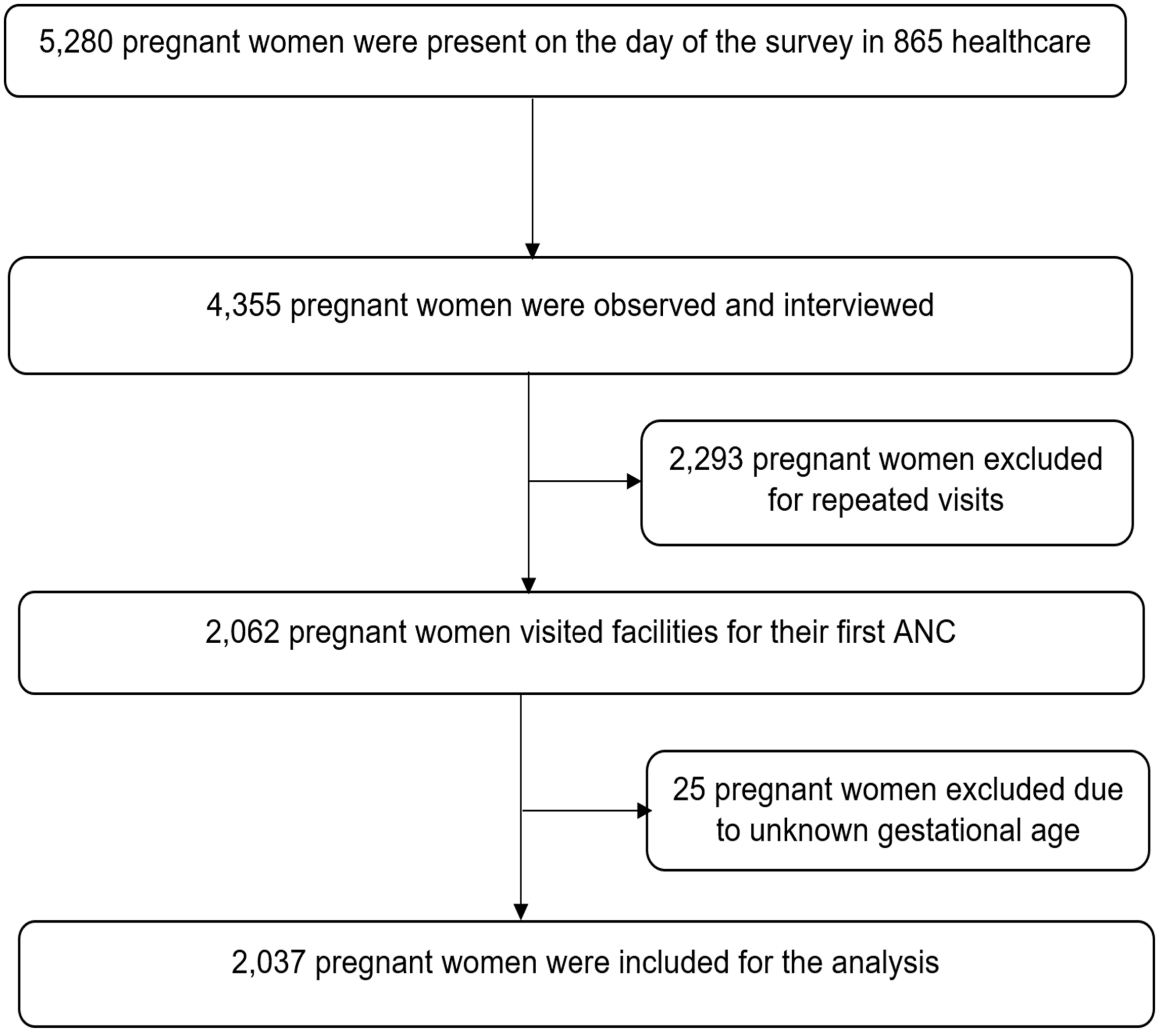
Flowchart showing the study participants’ selection process.

A total of 5,280 pregnant women attended 865 healthcare facilities for ANC on the day of the survey. The selection of pregnant women for interviews and observations was based on the anticipated ANC attendance at each facility on the survey day. However, a predetermined rule was put in place to limit the observation to a maximum of five clients per clinician, and up to 15 clients per healthcare facility. Interviews were conducted exclusively with pregnant women whose consultations had been observed before they left the facility. Based on these criteria, the consultations of 4,355 pregnant women were observed, and these women were subsequently interviewed. After excluding 2,293 pregnant women who were attending for repeat visits and an additional 25 due to unknown gestational age, a total of 2,037 pregnant women were included in the final analysis (Fig 1) [37].

### Variables of interest

#### Dependent variable.

The dependent variable was timely ANC initiation, defined as attending the first ANC visit within the first 12 weeks of pregnancy. This variable was coded as 1 for timely ANC initiation and 0 otherwise [14].

#### Independent variables.

The study assessed 14 independent variables, selected through a comprehensive review of online literature on their impact on timely ANC in previous studies [21,38–40]. The individual-level variables were maternal age, marital status, educational status, gravidity, number of births, number of living children, healthcare insurance coverage, area of residence, partner involvement, knowledge of signs of pregnancy complications, and healthcare facility's distance from home. The facility-level variables were region, type of healthcare facility, and facility ownership. The categorization and descriptions of all independent variables for this study are presented in Table 1.

**Table 1:** Description of independent variables for factors associated with timely ANC initiation in Ethiopia, ESPA 2021/2022.

Variable	Description
Individual level variable	
Maternal age	Maternal age (in years) was categorized as ‘1’ = 12–24, ‘2’ = 25–34, and ‘3’ = 35 years and above
Marital status	Categorized as ‘1’ = married and ‘2’ = other (single, widowed, divorced, and separated).
Educational status	Categorized as ‘1’ = never attended school, ‘2’ = primary education, ‘3’ = secondary education, and ‘4’ = higher education.
Gravidity	Referred the total number of pregnancies over women’s lifetime, was categorized as ‘1’ = primigravida (first pregnancy) and ‘2’ = multigravida (more than one pregnancy).
Number of births	Referred to the total number of births over a woman’s lifetime, it was categorized as ‘1’ = nulliparous (did not give birth), ‘2’ = Parus (gave birth of at least one)
Number of living children	Categorized as ‘1’ = 0, ‘2’ = 1–2, ‘3’ = 3 or more
Healthcare insurance coverage	Categorized as ‘1’ = yes and ‘2’ = no or don’t know
Area of residence	Coded as ‘1’ = urban and ‘2’ = rural
Partner involvement	Categorized as ‘1’ = No (never) and ‘2’ = Yes (always or sometimes)
Healthcare facility distance from home	Coded as ‘1’ = Yes (the facility was the nearest available facility to the woman’s home) and ‘2’ = No (the facility was not the nearest available facility to the woman’s home)
Knowledge of signs of pregnancy complications	Women were coded as ‘1’ (not knowledgeable) if they could not identify at least three of the following warning signs of pregnancy complications: vaginal bleeding, swollen hands or face, headache or blurred vision, fever, fatigue or breathlessness, seizures or convulsions, and reduced or absent fetal movements; otherwise, they were coded as ‘2’ (knowledgeable)
Facility level variable	
Regional location	Referring to the regional locations of the facilities where women attended ANC, coded as ‘1’ = Metropolis (Addis Ababa, Dire Dawa and Harari), ‘2’ = Large central (Amhara, Oromia, South Nations, Nationalities, and Peoples regions (SNNP), Sidama), ‘3’ = Small peripheral (Afar, Benishangul-Gumuz, Gambella, somali) [41]
Type of healthcare facility	Categorized as ‘1’ = Hospital (referral, general, primary), ‘2’ = Health centre, ‘3’ = Clinic (higher, medium, lower, specialty), and ‘4’ = Health Post
Facility ownership	Categorized as ‘1’ = Public and ‘2’ = Other (military, prison, private-for-profit, mission/faith-based, non-profit)

### Statistical analysis

All analyses were performed using Stata version 16 (StataCorp, College Station, TX, USA), incorporating client weights (c005/1,000,000) and the (svy) command to adjust for the complex and disproportionate sampling design. Descriptive statistics of participants’ background characteristics were presented as frequencies and percentages. We used multilevel mixed-effects logistic regression to examine factors associated with timely ANC initiation, as the hierarchical structure of SPA data violates standard logistic regression assumptions, particularly the assumptions of independence and constant variance. For instance, pregnant women (level one) were nested within healthcare facilities (level two), potentially leading to correlated patterns of timely ANC initiation among women within the same facility. In this case, multilevel mixed-effects logistic regression allows for more accurate estimation of the associations between independent variables at both levels and the outcome variable, by accounting for potential interactions within and between levels.

A total of four models were fitted, incorporating both fixed and random effects. The first model, the null model, included only a random intercept with no predictors. Model I examined the effects of individual-level variables on timely ANC initiation, whereas Model II assessed the contribution of facility-level factors. The final model, Model III, integrated both individual- and facility-level variables to evaluate their combined influence on timely ANC initiation.

Random effects analyses in the null model, conducted to quantify variation in timely ANC initiation across facilities, were presented using the Intra-class Correlation Coefficient (ICC) and Median Odds Ratio (MOR). The ICC, which quantifies the variation in timely ANC initiation across healthcare facilities and represents the proportion of total variation attributable to facility-level differences (Vf), is calculated as ICC = (Vf/ (Vf + 3.29)) × 100. The MOR expresses the median odds of timely ANC initiation when comparing a randomly selected client from a facility with higher rates to one from a facility with lower rates, and is calculated as MOR = exp (0.95 √Vf) [42].

Fixed-effect analyses in the subsequent models were conducted to assess the associations of individual- and facility-level independent variables with timely ANC initiation, with results presented as adjusted odds ratios (AOR) and 95% confidence intervals (CI), and statistical significance considered at p-value < 0.05. We employed the Variance Inflation Factor (VIF) to assess potential multicollinearity among the independent variables, and all values fell within the acceptable threshold (maximum VIF = 2.58, minimum VIF = 1.01, mean VIF = 1.35). Finally, models were compared using the deviance = −2 (log-likelihood) and Akaike’s Information Criterion (AIC), and the model with the lowest values for both metrics was selected as the best-fitting model.

### Ethics approval and consent to participate

Since anonymised data were used with permission from the DHS program and ethical approval from the Human Research Ethics Committee at the University of New South Wales (HC230398) and the Ethiopian Midwives Association (EMwA-IRB-SOP/011/4-24), obtaining informed consent from the participants was not required. All ESPA Survey protocols were strictly followed, the dataset was handled with the highest level of confidentiality.

## Results

### Characteristics of the pregnant women and healthcare facilities

This study included a total of 2,037 pregnant women attending their first ANC visit for their most recent pregnancy. The majority of women were aged 12–24 years (50.5%; n = 1,020), resided in rural areas (57.8%; n = 1,177), were married (97.3%; n = 1,982), had experienced more than one pregnancy (60.7%; n = 1,237),and had no living children (43.4%; n = 881). Among the women, 30.5% (n = 620) had no formal education, 90.4% (n = 1,842) lacked knowledge of the signs of pregnancy complications, and 65.6% (n = 1,334) of partners were not involved in their ANC. Most women received ANC in public facilities (89.9%; n = 1,831), and 52.2% (n = 1,063) received care at hospitals (Table 2).

**Table 2:** Background characteristics of pregnant women and healthcare facilities in Ethiopia, 2021/2022.

Variable	Frequency	Percentage
Individual level variable		
Age (years) (n = 2,020)		
12–24	1,020	50.5
25–34	913	45.2
35+	87	4.3
Marital status (n = 2,037)		
Married	1,982	97.3
Other	55	2.7
Education status (n = 2,033)		
No education	620	30.5
Primary school	712	35
Secondary school	510	25.1
Higher degree education	191	9.4
Gravidity (n = 2,037)		
Primigravida	800	39.3
Multigravida	1,237	60.7
Number of births (n = 2,029)		
Nulliparous	860	42.4
Parus	1,169	57.6
Covered by healthcare insurance (n = 2,036)		
Yes	683	33.5
No	1,353	66.5
Area of residence (n = 2,037)		
Urban	860	42.2
Rural	1,177	57.8
Partner’s involvement (n = 2, 035)		
No	1,334	65.6
Yes	701	34.4
Number of living children (n = 2,029)		
0	881	43.4
1–2	753	37.1
3+	395	19.5
Knowledge of signs of pregnancy complications (n = 2,037)		
Not Knowledgeable	1,842	90.4
Knowledgeable	195	9.6
Healthcare facility nearest to home (n = 2,036)		
Yes	1,683	82.6
No	354	17.4
Facility level variable		
Region (n = 2,037)		
Metropolis	159	7.8
Large central	1685	82.7
Small peripheral	193	9.5
Type of healthcare facility (n = 2,037)		
Hospital	1,063	52.2
Health centers	568	27.9
Clinics	120	5.9
Health post	286	14.0
Facility ownership (n = 2,037)		
Public	1,831	89.9
Other	206	10.1

### Magnitude of timely antenatal care initiation in Ethiopia

**Fig 2:**
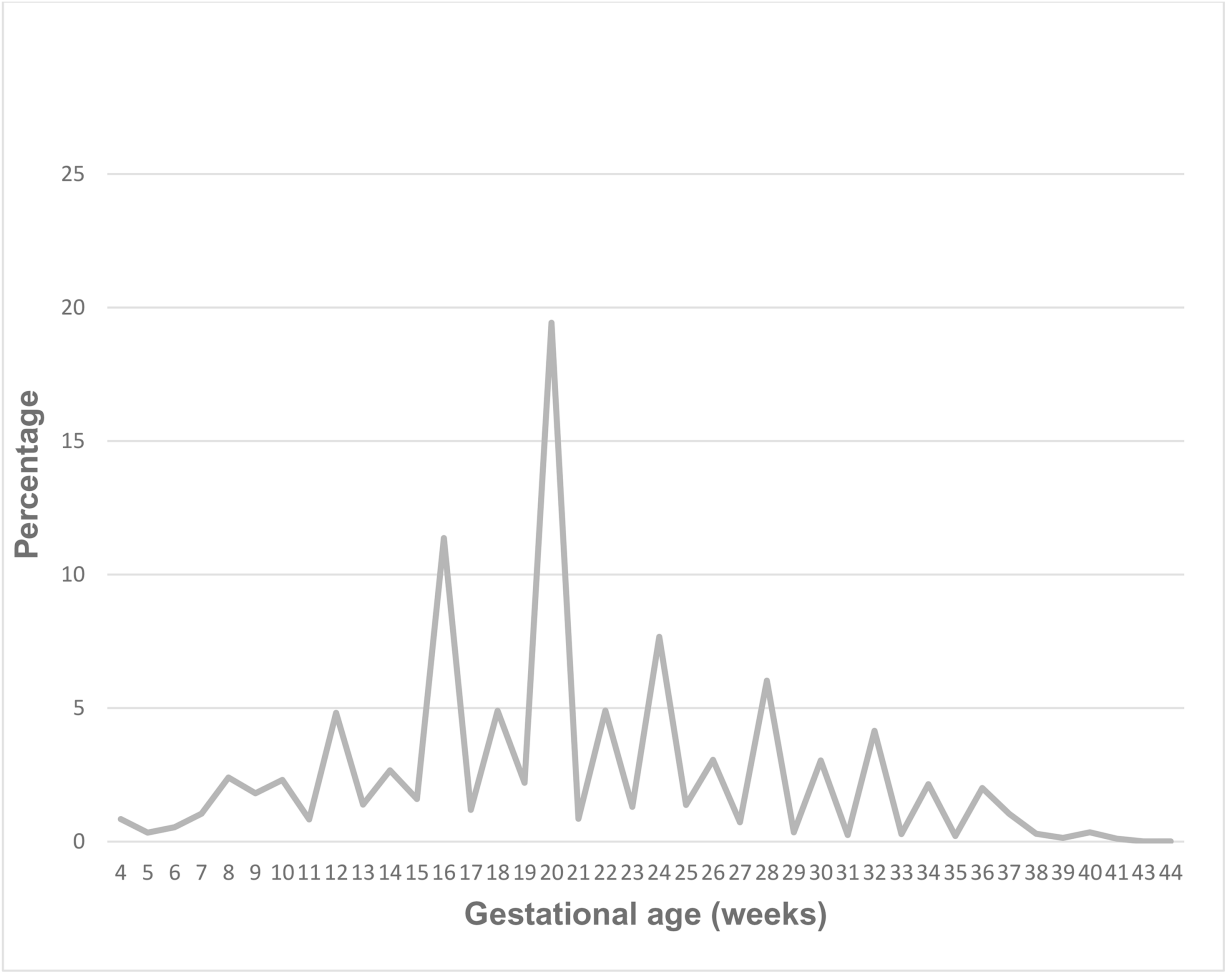
The percentage distribution of pregnant women by gestational age at ANC initiation in Ethiopia.

The proportion of pregnant women in Ethiopia who initiated ANC within the first 12 weeks of pregnancy for their most recent pregnancy was 14.9% (95% CI: 10.2, 21.5). The mean gestational age at which the women-initiated ANC was 22 weeks, ranging from 4 to 44 weeks. The percentage distribution of participants by gestational age at ANC initiation is shown in Fig 2.

### Results of random effects and model fitness

In the null model, the ICC of 0.35 indicates that 35% of the variation in timely ANC initiation was attributable to unobserved differences between healthcare facilities. This between-facility variation remained at 35% in Model I, which included only individual-level variables (ICC = 0.35). In the facility-level model (Model II), the ICC decreased to 25% (ICC = 0.25) and decreased to 24% in the final model (Model III) (ICC = 0.24). This highlights those variations in timely ANC initiation were influenced by both individual and healthcare facility level factors. In addition, the MOR value of 3.55 in the null model indicates that, when selecting a client at random, one from a facility with lower clients’ timely ANC initiation and another from a facility with higher clients’ timely ANC initiation, those in the facility with clients’ lower timely ANC initiation had 3.55 times lower odds of timely ANC initiation than those in the facility with higher clients’ timely ANC initiation. The final model (Model III), including individual and facility level variables, showed the lowest AIC and deviance, indicating the best model fit (Table 3).

**Table 3:** Comparison of models and random-effects analysis of timely antenatal care initiation in Ethiopia.

Parameter	Null model	Model I	Model II	Model III
VF	*1.78*	*1.77*	*1.07*	*1.06*
ICC	*0.35*	*0.35*	*0.25*	*0.24*
MOR	3.55	3.54	3.54	2.66
Model fit				
LLR	*−757.95*	*−724.28*	*−730.51*	*−699.79*
Deviance	*1515.89*	*1448.56*	*1461.02*	*1399.58*
AIC	*1519.89*	*1476.557*	*1477.02*	*1439.58*

### Individual and facility level factors associated with timely antenatal care initiation in Ethiopia

Table 4 shows the fixed-effects estimates of factors associated with timely ANC initiation across the three models (Model I–III). The multivariable multilevel mixed-effects logistic regression analysis, which simultaneously considered both individual and facility level factors, showed that maternal education, partner involvement, distance to the healthcare facility, the facility’s region, and its ownership were significantly associated with timely ANC initiation among pregnant women (p < 0.05).

**Table 4:** Determinants of timely antenatal care initiation at individual and facility level in Ethiopia: fixed-effects multilevel analysis.

Variable	Model I	Model II	Model III
	AOR (95% CI)	AOR (95% CI)	AOR (95% CI)
Individual level variable			
Age (years)			
12–24	1.00		1.00
25–34	1.14 (0.78, 1.65)		1.01 (0.70, 1.45)
35+	1.21 (0.54, 2.70)		0.94 (0.43, 2.08)
Marital status			
Married	1.00		1.00
Other	0.47 (0.13, 1.72)		0.45 (0.12, 1.61)
Education status			
No education	1.00		1.00
Primary school	**1.92 (1.17, 3.14)**		**1.74 (1.08, 2.82)**
Secondary school	**1.83 (1.09, 3.08)**		1.57 (0.95, 2.62)
Higher degree education	**2.12 (1.18, 3.82)**		1.65 (0.92, 2.96)
Gravidity			
Primigravida	1.00		1.00
Multigravida	1.46 (0.69, 3.11)		1.44 (0.69, 2.98)
Number of births			
Nulliparous	1.00		1.00
Parus	0.67 (0.14, 3.25)		0.67 (0.15, 3.14)
Number of living children			
0	1.00		1.00
1-4	0.65 (0.15, 2.79)		0.69 (0.17, 2.83)
5+	0.49 (0.11, 2.22)		0.56 (0.13, 2.47)
Partner’s involvement			
No	1.00		1.00
Yes	**1.56 (1.13, 2.16)**		**1.42 (1.03, 1.96)**
Healthcare facility nearest to home			
Yes	1.00		1.00
No	**0.51 (0.35, 0.75)**		**0.44 (0.30, 0.64)**
Facility level variable			
Region			
Metropolis		1.00	1.00
Large central		**0.24 (0.15, 0.38)**	**0.24 (0.15, 0.40)**
Small peripheral		**0.24 (0.13, 0.46)**	**0.27 (0.13, 0.52)**
Type of healthcare facility			
Hospital		1.00	1.00
Health centers		1.03 (0.71, 1.50)	0.99 (0.67, 1.45)
Clinics		1.32 (0.70, 2.51)	1.30 (0.68, 2.50)
Health post		0.71 (0.41, 1.25)	0.69 (0.39, 1.22)
Facility ownership			
Public		1.00	1.00
Other		**2.37 (1.42, 3.96)**	**2.49 (1.45, 4.28)**

Pregnant women who had attended primary school were 1.74 times more likely to initiate ANC timely compared to those who had never attended school (AOR = 1.74, 95% CI: 1.08, 2.82). The odds of timely ANC initiation were 1.42 times higher among pregnant women whose partners accompanied them to the healthcare facility for ANC compared to those whose partners did not accompany them during their visit (AOR = 1.42, 95% CI: 1.03, 1.96). Women who did not visit nearby healthcare facilities for ANC had 56% lower odds of timely ANC initiation compared to women who visited nearby healthcare facilities for ANC (AOR = 0.44, 95% CI: 0.30, 0.64).

The odds of timely ANC initiation were 76% times lower among women who sought ANC from healthcare facilities in large central regions (AOR = 0.24, 95% CI: 0.15, 0.40) and 73% times lower among those who sought ANC from facilities in small peripheral regions (AOR = 0.27, 95% CI: 0.13, 0.52), compared to women who sought ANC from facilities in the metropolis. Women who sought ANC at non-public healthcare facilities had 2.49 times higher odds of initiating ANC timely compared to those who sought ANC at public healthcare facilities (AOR = 2.49, 95% CI: 1.45, 4.28).

## Discussion

To our knowledge, this is the first nationwide, facility-based study examining the timing of ANC initiation and its determinants among women at their first ANC visit for their most recent pregnancy in Ethiopia. Our findings revealed that, on average, pregnant women begin ANC at approximately 22 weeks of pregnancy, with only 14.94% initiating within the first 12 weeks, as recommended by the WHO. Pregnant women’s educational status, partner involvement, distance to the healthcare facility, the facility’s regional location, and its ownership were identified as factors influencing the timing of ANC initiation.

The WHO advises pregnant women to begin ANC within the first 12 weeks of pregnancy, facilitating early disease prevention, diagnosis, and treatment, while promoting compliance with recommended visits and interventions throughout pregnancy, thereby improving pregnancy outcomes [14]. However, this study showed that 14.94% of pregnant women in Ethiopia initiated ANC within the recommended timeline. This finding is consistent with studies conducted in parts of the country, which reported 14.33% [43], but lower than that reported in a nationwide study in the country, which reported 37.7% and to which two of the current study’s authors contributed [26]. The divergence among the studies may be due to differences in study setting and ANC timing measurement. Our study and the study conducted in parts of the country were facility-based and recorded timing in weeks during service provision, whereas the previous nationwide study was community-based and relied on women’s recall in months, potentially overestimating timely initiation, as noted in previous research [20]. This finding highlights the need for interventions to ensure timely ANC initiation in Ethiopia, as delays among most women may impede progress in reducing maternal and neonatal mortality in line with SDG-3

The study revealed that the average gestational age at which women begin ANC is 22 weeks. This is consistent with findings from a study conducted in the same country by Gebremeskel et al. [44], but later than reports from Ghana, where women initiated ANC at an average of 12 weeks [45]. The observed variation may reflect lower levels of women’s literacy, wealth, media exposure, internet use, and employment, along with limited healthcare access and provider availability in Ethiopia compared to Ghana, as reported in previous studies [46–49]. Furthermore, variations in these factors across regions in Ethiopia [46] could explain the significant disparities observed in the current study, where women attending facilities in large central and small peripheral regions were less likely to initiate ANC timely compared to those in the metropolis regions. Women’s education, media exposure, internet use, employment, and access to well-staffed healthcare facilities are undeniably among the pivotal factors contributing to timely ANC initiation [20,29]. It is believed that access to well-staffed healthcare facilities could alleviate women’s burdens from long distance travel and financial constraints, while education, media exposure, and internet use could improve awareness of when and where to seek care, and employment could further enhance women’s financial autonomy and healthcare decision-making. Similarly, this study revealed that pregnant women with primary education were more likely to initiate ANC timely than those who had never attended school

The finding of this study corroborates evidence from a study conducted in Afghanistan, which showed that women who attended ANC with their partners were more likely to initiate ANC timely than those who attended alone [50]. This may be explained by the fact that partners who accompany their wives to ANC tend to have higher education, supportive attitudes toward ANC, awareness of ANC and pregnancy-related risks, recognition of ANC as a shared responsibility, and engagement in joint decision-making, which collectively support and facilitate their wives’ timely ANC initiation [51,52]. Consistent with this, a study in Ethiopia reported that when partners were involved in ANC decisions, their wives were more likely to initiate care on timely [53]. Partner involvement is especially important for promoting timely ANC initiation in SSA, including Ethiopia, as women’s access to care often depends on their partners’ approval due to financial dependence and prevailing cultural norms [54,55]. However, research on partner involvement in ANC initiation in Ethiopia remains limited, highlighting the need for further investigation.

Our study showed that women who bypassed nearby healthcare facilities for ANC were less likely to initiate ANC timely compared to those who utilised nearby facilities. This could be because of the need to travel longer distances, incur higher out-of-pocket expenses, and spend more time accessing care than women who attended nearby facilities [56,57]. A qualitative study conducted in Cameroon similarly reported that distance, cost, and time constraints are major barriers to timely ANC initiation [58]. Evidence from the country also indicates that bypassing the nearest healthcare facility is associated with women’s illness status and their negative perceptions of the quality of care provided [59]. While bypassing may allow women to access care they perceive as higher quality, it can inadvertently delay ANC initiation, as women may wait until they consider their condition serious enough to visit their preferred facility. This underscores the need to strengthen the quality of care in primary public healthcare facilities, such as health centres and health posts, as these facilities are accessible to a large proportion of the population in the country, and calls for further research into women’s decision-making around facility choice and its implications, particularly for ANC uptake.

In this study, non-public healthcare facilities were more likely to have women initiating ANC in a timely manner compared to public healthcare facilities. This may be due to the relatively better availability of essential supplies, equipment such as ultrasound, and more supportive, respectful, and patient-centred care in non-public healthcare facilities compared to public ones [54,60]. These factors may increase women’s confidence in the facilities, satisfaction, and perceived quality of services, which likely encourages them to initiate ANC in a timely manner. A facility-based study conducted in Uganda to examine determinants of timely ANC also found similar results [38]. Since non-public healthcare facilities in Ethiopia primarily serve urban communities with higher socioeconomic status [61], addressing potential gaps in public healthcare facilities is essential to make public facilities every woman’s preferred choice for ANC, similar to non-public healthcare facilities, which would improve timely ANC initiation in the country.

### Strengths and limitations of the study

This study had the following strengths. First, it utilised data from the most recent nationwide facility-based survey (ESPA), in which the timing of ANC initiation was recorded in weeks, ensuring reliable estimates and making the findings applicable across Ethiopia. Second, it employed multilevel modelling, which accounted for the nested structure of the SPA data and enabled the examination of predictors of timely ANC initiation at both individual and facility levels. However, it is important to acknowledge the following limitations. First, the survey did not include one region of the country (Tigray), so the findings may not be representative of that region. In addition, the cross-sectional design limits the analysis to associations and does not allow for causal inferences between the explanatory and outcome variables.

## Conclusions

Nearly one in seven pregnant women in Ethiopia initiated ANC within the first 12 weeks of pregnancy, far below the WHO target, which recommends that all pregnant women should initiate ANC within this period. Timely ANC initiation was more likely among pregnant women with primary education, those whose partners were involved, and those attending non-public facilities, whereas bypassing nearby facilities or attending facilities located in large central or small peripheral regions reduced the likelihood of timely initiation. Thus, greater emphasis should be placed on raising community awareness about the importance of timely ANC initiation, promoting education and partner involvement, and encouraging women to use nearby available healthcare facilities to improve timely ANC initiation in Ethiopia. In addition, improving healthcare access and provider availability, especially in the large central and small peripheral regions, ensuring adequate resources for ANC, and promoting supportive and respectful care in public healthcare facilities would help to reduce facility-related barriers that may have prevented women from initiating ANC timely. Future studies should qualitatively explore factors influencing the timing of ANC initiation and conduct experimental research aimed at increasing partner involvement in ANC in Ethiopia

## Abbreviations

AIC: Akaike’s Information Criterion
ANC: Antenatal care
AOR: Adjusted Odds Ratio
CI: Confidence Interval
DHS: Demographic and Health Survey
ESPA: Ethiopian Service Provision Assessment
ICC: Intra-class Correlation Coefficient
LLR: Log Likelihood Ratio
LMICs: low-income countries
MOR: Median Odds Ratio
SDGs: Sustainable Development Goals
SNNP: South Nations, Nationalities, and Peoples regions
SSA: Sub-Saharan Africa
VIF: Variance Inflation Factor
WHO: The World Health Organization
